# Nasal Carriage Rate of Biofilm Producing Methicillin Resistant *Staphylococcus aureus* and Its Associated Factors Among Health Care Workers at Hospital of Central Ethiopia

**DOI:** 10.1002/mbo3.70266

**Published:** 2026-03-15

**Authors:** Wubeshet Mathewos, Abera Kumalo, Takele Teklu, Tigistu Demisse, Muluneh Temesgen, Tariku Chinasho

**Affiliations:** ^1^ School of Medical Laboratory, College of Health Sciences and Medicine Wolaita Sodo University Sodo Ethiopia; ^2^ Department of Medical Laboratory Science Durame General Hospital Durame Ethiopia; ^3^ Department of Medical Laboratory Science Hosanna Health science College Hosanna Ethiopia; ^4^ 11700 Old Columbia pike, #1411 Silver Spring Maryland USA

**Keywords:** associated factors, biofilm producing MRSA, Ethiopia, health care workers, MRSA

## Abstract

Not susceptible to methicillin *Staphylococcus aureus* (MRSA), is a potentially harmful bacteria that is resistant to the most important antimicrobial agents. Because MRSA is so resistant to many antibiotics, it can cause illnesses by forming biofilms. The aim of this study was to assess the nasal carriage rate of biofilm‐producing methicillin‐resistant *Staphylococcus aureus* (MRSA) and its associated factors among HealthCare Workers at Wachemo University Nigist Ellen Mohammed Memorial Comprehensive Specialized Hospital, Central Ethiopia. This cross‐sectional study, carried out at Wachemo University Nigist Ellen Mohammed Memorial Comprehensive Specialized Hospital, Central Ethiopia from August 1 to November 30, 2023. Nasal swab samples from 294 healthcare workers (HCWs) were obtained using sterile cotton swabs. Bacterial isolates were identified using standard culture methods on Mannitol Salt and Blood Agar, while antimicrobial susceptibility testing and biofilm formation assessments followed the CLSI 2023 (M100, 33rd edition) guidelines via the Kirby‐Bauer disk diffusion methods. All laboratory analyses were performed in triplicate to ensure consistency. Data were double‐entered into Epi Data version 4.6 and cross‐checked for accuracy. Missing or inconsistent data were verified against original laboratory records and latterly then, exported to SPSS V25 for analysis. Descriptive statistics and logistic regression were applied for statistical evaluation, with a *p*‐value of ≤ 0.05 regarded as statistically significant. In this study, the occurrence rates of S. aureus, MRSA, and biofilm‐producing MRSA were 98 out of 294 isolated strains (33.4%), 41 out of 294 isolated strains (13.9%), and 28 out of 294 isolated strains (9.5%), respectively. The MRSA strains exhibited high sensitivity to linezolid, rifampicin, and vancomycin while showing resistance to cefoxitin, cotrimoxazole, and ciprofloxacin. A history of prior hospitalization (length of stay in the hospital) was statistically significant for the colonization of biofilm‐producing MRSA, with an adjusted odds ratio of 10.00 (95% CI: 1.36–73.3; *P* = 0.024). MRSA and MRSA that produces biofilms were found to be 41.8% and 68.3% prevalent overall in the study area, respectively. Biofilm‐producing MRSA is a potential cause of healthcare‐associated diseases. Therefore, these findings emphasize the urgent need for improved infection‐prevention practices and routine screening of healthcare workers to mitigate the risk of healthcare‐associated infections.

## Graphical abstract

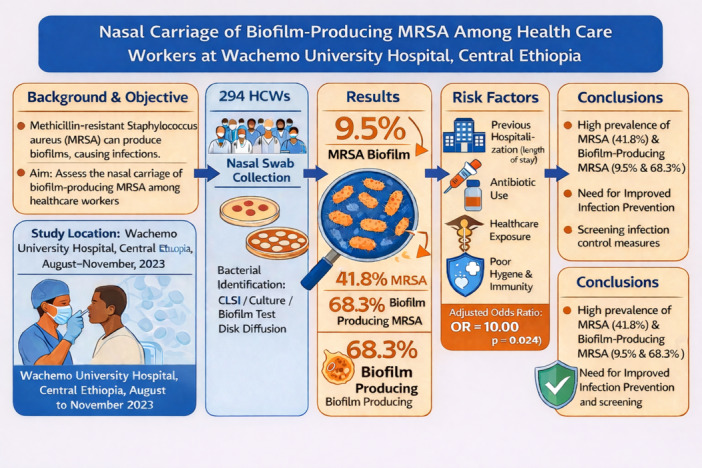

## 1 Introduction

*Staphylococcus aureus* is a spherical, gram‐positive, catalase and coagulase‐producing bacterium that commonly colonizes the skin and nasal mucosa of healthy individuals worldwide (Firouzjaei et al. 2024). Although it is a normal commensal organism, *S. aureus* is an opportunistic pathogen capable of causing a wide spectrum of infections, ranging from folliculitis and food‐borne intoxications to life‐threatening conditions such as endocarditis, osteomyelitis, and septicemia. Its remarkable ability to survive in the environment and adhere to the surfaces of medical devices has made this bacterium one of the leading causes of hospital‐acquired infections among both patients and healthcare workers (Malachowa and DeLeo 2010; Kpeli et al. 2016).

Methicillin‐resistant *S. aureus* (MRSA) is a new and continuously emerging strain that is one of the earliest medically significant staphylococci, is potentially harmful, and resistant to certain antibiotics (Foster 2002). Due to the fact that unidentified carriers can spread MRSA isolates within healthcare settings, hospitalized patients and health workers are indirectly contaminated and develop a serious risk (Control CfD 2014; Lakhundi and Zhang 2018). MRSA colonization is a common threat to infections in people of all ages. It is particularly known for people who come into contact with MRSA in hospital environments, where the chance of contracting an infection is approximately 30% (Lowy 1998; Abimana et al. 2019). During hospital stays, transfers, or other healthcare‐related interactions, forms of MRSA can spread through direct skin‐to‐skin contact (Foster 2002).

Globally, MRSA is an important pathogen in hospitals that causes significant illness and death (Haddadin et al. 2002). It has become one of the most prevalent causes of healthcare‐associated infections and is still a major cause of poor management practices (Mehta et al. 1998). Healthcare workers working at the hospital–community interaction may act as asymptomatic carriers of the pathogen and transmit infection in community and hospital settings (Albrich and Harbarth 2008). These microbes are MDR and resistant to three or more antibacterial groups, and their concerning global proportions have been reported (Choi et al. 2006).

Biofilm‐producing bacteria are found everywhere and can cause major problems in a variety of settings, including hospital environments. Compared to their free‐swimming counterparts, bacteria living in biofilms show more than a 1000‐fold increase in antibiotic tolerance (Simmons 2011).

The emergence of MRSA due to its biofilm‐producing ability, which often leads to multi‐drug resistance (MDR), renders the treatment of MRSA infections more challenging (Murugan et al. 2016). Presently, MRSA strains that produce biofilms are widespread and resistant to the majority of antibiotic categories, including β‐lactams, steroids, macrolides, and quinolones (Coia et al. 2006). Biofilm‐forming MRSA is clinically significant owing to its high antimicrobial resistance and enhanced pathogenicity (Blanco‐Cabra et al. 2021). Compared to diseases caused by other MRSA strains, those caused by biofilm‐forming MRSA strains are linked to extended hospitalizations, greater expenses, and delayed administration of antibiotics.

The MDR ability of MRSA has been linked to improper antibiotic use and/or noncompliance with institutional guidelines. Similarly, the regulatory system of healthcare facilities in developing countries is weak due to a lack of adequate resources, poor adherence to infection‐prevention measures, overcrowding, along with elevated patient flow, contamination by others between medical professionals and patients, and a higher rate of inappropriate treatment with empirical antibiotics. This gap creates a challenge for hospital leaders during the planning of appropriate antibiotics and for health workers in prescribing appropriate antibiotics for their patients, which in turn results in a high patient's financial burden of hospitalization. This problem is becoming serious in a setting where second third‐line antibiotics are expensive and even not accessible in developing countries, particularly in Ethiopia (Khatoon et al. 2018).

Several studies have assessed the magnitude of MRSA and its risk factors in Ethiopia. The studies reported a distinct magnitude of MRSA of 49.7%, 2.4%, and 17.5%. Furthermore, the evidence revealed confounding risk factors, used nasal specimens, and studied a few study areas (Gebremedhn et al. 2016; Dilnessa and Bitew 2016; Tadesse et al. 2018).

Infectious diseases caused by biofilm‐producing MRSA continue to plague sub‐Saharan Africa as a whole, with nosocomial infections accounting for far more of the disease burden than in developed countries (Mbim et al. 2016; Nejad et al. 2011). The extent of the problem of HAIs caused by biofilm‐producing MRSA in Central Ethiopia and the majority of African countries is grossly underestimated (Nejad et al. 2011; Eshetie et al. 2015). Unfortunately, the interaction between HCWs and the transmission of MDR pathogens among patients in the nasal carriage rate among healthcare workers is not well documented in Central Ethiopia, particularly in our study area (Eshetie et al. 2015). It is estimated that in resource‐constrained countries, 50% or more of hospitalized patients will develop a biofilm that produces MDR MRSA (Nejad et al. 2011). With the deterioration of healthcare infrastructure and falling healthcare standards in our study's public health sector (Akoru et al. 2016), it is critical that more attention be paid to the role of the hospital environment and HCW nasal carriage in the transmission of MDR nosocomial infections. Our study area was selected due to high patient flow, a large number of healthcare workers, and medical students who are enrolled in an educational institution. Regular screening of biofilm‐producing methicillin‐resistant *S. aureus* in HCWs could minimize the potential outbreak of the pathogen. In Central Ethiopia, in the case of Wachemo University Nigist Ellen Mohammed Memorial Comprehensive Specialized Hospital (WUNEMMCSH), no studies have been conducted to determine the prevalence of biofilm‐producing *S. aureus* among HCWs. Therefore, this study aimed to assess the nasal carriage rate of biofilm‐producing methicillin‐resistant *Staphylococcus aureus* among healthcare workers at Wachemo University Nigist Ellen Mohammed Memorial Comprehensive Specialized Hospital.

## 2 Materials and Methods

### 2.1 Study Area, Time Frame and Design

An institution‐based cross‐sectional study was conducted from August 1 to November 30, 2023, at WUNEMMCSH in Hosanna, Central Ethiopia. The town is 232 km away from Addis Ababa. The hospital provides services to an estimated four million people in the catchment area. The hospital is staffed with 984 healthcare workers with different qualifications.

### 2.2 Source Participants

Source participants were all healthcare workers during the study periods.

#### 2.2.1 Study Participants

The study participants were randomly selected healthcare workers from different departments.

### 2.3 Sample Size Determination and Sampling Techniques

#### 2.3.1 Sample Size Determination

To determine the sample size, a single‐population proportion formula was applied. The value of proportion (P) was received as 44.1% (0.441) in a previous study conducted at Dessie, Ethiopia, by Shibabaw et al. (2013). The following standard formula was used to determine the sample size, taking into account a 95% confidence interval and a 5% margin of error:

The sample size:

$$N=\frac{{\;Z}_{{\left(\frac{a}{2}\right)}^{2\;P(1-p)}}}{d^{2}}$$

$Z_{{\left(\frac{a}{2}\right)}^{2}}=\;$At 95% confidence interval Z value (*α* = 0.05) = 1.96

*p* = the proportion of occurrence of the event to be studied is 44.1% (0.441).

*d* = Margin of error at (5%) (0.05)

$$N=\frac{1.96^{2}x\;0.441(1-0.441)}{0.05^{2}}N=375$$

And also after adding 10% of non‐respondents' rates, there were 412 medical professionals in the group being studied.

Since the total population is less than 10,000, the correction formula is

$$\frac{n}{1+\frac{n}{N}}=\frac{412}{1+\frac{412}{984}}=294$$

where *n* is the required sample size and *n* is the total sample size. This is the standard formula for the required sample size that is obtained by the sample size correction because the total number of individuals is below ten thousand, and

*N* is the estimated number of individuals.

The sample size is 294.

#### 2.3.2 Sampling Techniques and Sampling Frame

Following the selection of HCWs for the study, a method of systematic random sampling was employed. Ten wards of interest included Medical, Surgical, and Pediatrics, Gynecology/Obstetrics, Laboratory, Out Patients Diagnosis (OPD), Pharmacy, Orthopedics, ICUs, and Emergency wards formed for strata for sampling. A total of 984 HCWs were identified from the selected wards. By considering this and using proportional allocation *n_i_* = *N_i_*/*N* *n =ni, ni were chosen by a systematic random sampling technique from each ward after proportional allocation was performed (Table 1). Similarly, 294 HCWs were chosen by a systematic random sampling method with every 3rd HCWs (by the formula *K* = *N*/*nf*, =984/294 = 3) from the selected wards. The first HCW is selected using the lottery method. Every third healthcare worker participated in this study.

**Table 1:** Sampling frame for the healthcare workers who were found at the selected department in WUNEMMCSH, Wachemo University Nigist Ellen Mohammed Memorial Comprehensive Specialized Hospital, Central Ethiopia, 2023.

Departments	Number of healthcare workers	
	Total (*N*_i_)	Sample (*ni*)
Medical	74	28
Pediatrics	98	33
Gynecology–Obstetrics	110	31
Laboratory	72	25
OPD	142	47
Pharmacy	58	21
Surgical	86	30
Orthopedics	78	22
ICUs	110	22
Emergency	156	35
Total	984	294

*Note:* where *ni* is the sample for the strata, *N*_i_ is the total population of each stratum, *N* is the sum of the total strata, *n* is the entire size of the sample, *K* is the sampling interval, and *N* is the total number of people.

### 2.4 Data Collection

Data from the HCWs in each ward were gathered through an organized questionnaire. The nostril brush samples were collected from HCWs before contact with the patient and after the one and a half hours cleaning of the wards during the daytime of the work (at the hours of 8:30 AM and 2:30 PM) are as follows, considering the fact that almost all HCWs exist.

### 2.5 Specimen Collection and Transportation

Nostril specimens from the patients were collected using sterilized cotton swabs. Following the study participants' sit‐down, the swabs were gently rotated on both nares at least four times for a period of 1 min. In the Hosanna Regional Health Institute Microbiology Laboratory, the swab tip was carefully put in a test tube with 1 mL of regular saline, sealed, and processed.

### 2.6 Laboratory Procedures

Mannitol Salt Agar (MSA) was used to inoculate and identify the isolates from nasal swabs, which were subsequently incubated aerobically for a period of 24 to 48 h at 37°C. The slide test method was used to conduct the catalase and coagulase tests after the plates had been incubated at room temperature (Cheesbrough 2005). The strain of *S. aureus* was identified as a colony that tested coagulase‐positive and was a mannitol fermenter (golden or cream color on MSA). And also, oxacillin was used to screen *S. aureus* isolates for cefoxitin opposition. Incubate the screen agar at 37°C for 18 to 24 h. Methicillin‐resistant colonies were defined as those that were within the area of inhibition zone but were smaller than the advised millimeter (Humphries et al. 2021).

#### 2.6.1 Detection of Biofilm Productions

The production of bacterial biofilm was detected using the tube method (TM). Sterile glass tubes containing tryptone soy broth were used. Loopful colony of each isolated strain was added to TSB tubes, which were then incubated for 24 h at 37°C. Each TSB test tube was filled with 2 mL of 2% glucose after 24 h, and the tubes were once more incubated for 24 h at the same temperature. The test tubes were twice aseptically cleaned with phosphate buffer saline at pH 7.4 to get rid of any unbound bacteria after the 48‐h incubation period was over. The tryptone soy broth growth media was then disposed of. Following phosphate buffer saline washing, 3 mL of 99% methanol was used to fix the remaining attached microorganisms, and the tubes were then left for 15 min. The methanol was then thrown away. It was decided to let the tubes dry. Three mL of 0.1% crystal violet was added to each test tube and left there for 5–6 min in order to observe the microorganisms' production of biofilm. The excess stain was removed from the test tubes by carefully washing them under running water. Following the removal of excess stain, the tubes were inverted and allowed to dry. By looking at the color of the tube's inner surface, the test results were deduced. Biofilm formation can be interpreted as positive when a visible film lines the wall and the bottom of the test tube. The amount of the biofilm formed was scored as weak/none, moderate, and high/strong. The experiment was performed in triplicate and repeated three times. The tubes were compared with control strains (Jain et al. 2013).

#### 2.6.2 Investigation of Antimicrobial Agents' Susceptibility

Using the Kirby‐Bauer disc diffusion method, which is advised by the Clinical and Laboratory Standards Institute, antimicrobial agents susceptibility testing was carried out (Cheesbrough 2005). Sterilized normal saline solution was used to emulsify the colonies, and suspension concentrations were compared until they matched the standard turbidity (McFarland, 0.5). Mueller‐Hinton agar was inoculated with the suspension (MHA The antimicrobial disks were placed on the surface of the agar and gently pressed down with sterile forceps. Then the medium was incubated at 37° C for 18–24 h. The results were reported as susceptible, intermediate, or resistant according to criteria developed in 2021 by Clinical and Laboratory Standards Institute (CLSI) (CLSI‐Clinical, Laboratory Standards Institute M 2023). Colonies that had an inhibition zone smaller than the advised millimeters were classified as “methicillin” resistant. The control, *Staphylococcus aureus ATCC 25923*, was employed (Cheesbrough 2005). Antibiotic disks containing Gentamicin 10 μg, Ciprofloxacin5 μg, Linezolid 300 μg, Cefoxitin 30 μg, Chloroampinicol 30 μg, Cotrimoxazole 25 μg, Tetracycline30 μg, Clindamycin 2 μg, Rifampicin 5 μg, Cloxacillin 5 μg, Vancomyin 30 μg, and Aiththromycin 15 μg were used. Antibiotic discs were selected according to the list of drugs for local availability, effectiveness, and specific isolated pathogen provided by CLSI (2023), and bacterial isolates, which are resistant to three or more classes of drugs, were considered as multidrug resistant (MDR) (Weinstein 2023).

### 2.7 Management of Data Quality

Quality control (QC) procedures were used throughout the laboratory work process to ensure the dependability of the investigation results. Every procedure, piece of equipment, and material was properly regulated. To reduce contaminants, hygienic practices were applied at every stage of collecting samples and inoculating into culture media. Every culture medium was made in compliance with the guidelines provided by the manufacturer. The incubation medium's accomplishment and sterility were examined. For every laboratory study, standard operating procedures (SOPs) were closely adhered to. The Ethiopian Public Health Institute (EPHI) provided the known bacterial strains, which included *Streptococcus pyogenes* (ATCC 49619), *Pseudomonas aerogenes* (ATCC 27853), and *Staphylococcus aureus* (ATCC 25923).

### 2.8 Data Management and Analysis

Data were brought into the Statistical Package for Social Sciences (SPSS) software version 25 for analysis after being entered into, verified, cleaned, and categorized for completeness in Epi version 4.6. The results of the study were described using descriptive statistics. Continuous data were outlined using means, while categorical variables were described using frequencies and percentages. The relationship between both dependent (Nasal carriage rate biofilm producing methicillin resistant *Staphylococcus aureus*) and independent variables (age, sex, professions of types, work experience, previous antibiotics use, use of mask, hand washing habit, Use of antiseptics for hand rub, types of antiseptics, prior hospitalization, diabetic mellitus) was investigated using logistic regression. First, binary logistic regression was used to examine the relationship between each independent and dependent variable. Multiple logistic regressions were used for variables with *p*‐values less than 0.25. The threshold for statistically significant differences was *p* ≤ 0.05. The strength of the correlation was described using the crude odds ratio (COR) and adjusted odds ratio (AOR). Lastly, tables, figures, and statements were used to present the findings.

### 2.9 Ethical Clearance

The studies involving humans were approved by Ethical Review Committee (ERC) of the College of Health Science and Medicine, Wolaita Sodo University by letter numbered with CHSM/ERC/07/2023. Then, Nigist Ellen Mohammed Memorial Comprehensive Specialized Hospital was officially granted permission to participate in the study through a letter of cooperation. After being fully informed about the study, the participants were asked to provide their informed consent to confirm their willingness to participate. To ensure privacy, every idea collected from study participants was encrypted and coded. The studies were conducted in accordance with the local legislation and institutional requirements.

## 3 Results

### 3.1 The Study Participants' Socio‐Demographic Characteristics

With a 100% response rate, 294 healthcare workers participated in the study. The mean age of those involved was 44, with a range of 25–64. Of the participants, 185 (62.9%) were between the ages of 25 and 34. Of the 185 women (62.9%), they were included. The average duration of employment was 9.8 years. Out of all the participants, the majority (119 or 40.5%) were nurses, followed by midwives, physicians, lab professionals, and pharmacists. The outpatient department 47 (16%) was the most common location for health professionals to work, followed by the emergency department 35 (12%) and the pediatric department 33 (11.2%) (Table 2).

**Table 2:** Socio‐demographic characteristics of healthcare providers at WUNEMMCSH, Central Ethiopia, 2023.

Variables	Number	Percentage (%)
Sex		
Male	109	37.1
Female	185	62.9
Age group		
25–34	185	62.9
35–44	65	22.1
45–54	39	13.3
55–64	5	1.7
Work experience in years		
> 1/2–5	149	50.6
6–10	45	15.3
11–20	66	22.5
21–30	28	9.5
> 30	6	2.1
Profession		
Medical doctor	61	20.7
Nurse	119	40.5
Midwife	68	23.1
Laboratory	25	8.5
Pharmacy	21	7.1
Working site		
Medical	28	9.5
Surgical	30	10.2
Pediatric	33	11.2
Gynecology‐obstetrics	31	10.5
Laboratory	25	8.5
Outpatient department	47	16.0
ICU	22	7.4
Pharmacy	21	7.1
Emergency department	35	12.0
Orthopedic	22	7.4
Use of antibiotics before one week of swab collection		
Yes	103	35.1
No	191	64.9
Use of Mask		
Yes	240	81.6
No	54	18.4
Hand washing habit after and before patient attendance		
Always	160	54.4
Usually	89	30.3
Not at all	45	15.3
Use of antiseptics for hand rub		
Always	152	51.7
Usually	111	37.8
Not at all	31	10.5
Antiseptics used after and before patient touch		
70% Alcohol only	128	43.5
Sanitizer only	107	36.4
Both	59	20.1
Prior hospitalization		
Yes	64	21.8
No	230	78.2
Diabetic mellitus		
Yes	37	12.6
No	257	87.4

### 3.2 Prevalence of *Staphylococcus aureus*

Of the 294 nasal swabs collected from healthcare providers and analyzed by culture, 98 were positive for *Staphylococcus aureus*, yielding an overall prevalence of 33.4% (98/294; 95% CI: 28.1%–38.7%).

### 3.3 Prevalence of MRSA

After identification and confirmation of *Staphylococcus aureus* isolates, the MRSA positivity rate was 41/294 (13.9%; 95% CI: 12.77–15.1). Among the 98 *S. aureus* isolates, 57 (58.2%) were methicillin‐sensitive *Staphylococcus aureus* (MSSA).

### 3.4 Prevalence of Biofilm‐Forming MRSA Isolates From Healthcare Workers

In vitro biofilm producers among the MRSA recovered from healthcare workers were 28/41(68.3%), followed by MSSA, 27/57(47.4%). Among in vitro biofilm producers among healthcare workers, MRSA was the most predominant biofilm producer isolate, which overall accounted for 28/41(68.3%), followed by MSSA, 27/57(47.4%). MRSA was the predominant biofilm producer compared with MSSA (Table 3).

**Table 3:** Prevalence of biofilm‐forming MRSA isolates from healthcare workers at WUNEMMCSH 2023.

Bacteria isolates	Biofilm production		Total	SD
	Biofilm producer	Non‐biofilm producer		
MRSA	28(68.3%)	13(31.7%)	41(41.8%)	0.45
MSSA	27(47.4%)	30(52.6%)	57(58.2%)	0.48
Total	55(56.1%)	43(43.9%)	98(100.0%)	0.47

Abbreviations: MRSA, methicillin‐resistance *Staphylococcus aureus*; MSSA, methicillin‐sensitive *Staphylococcus aureus*; SD, standard deviation.

### 3.5 Investigation of Antimicrobial Agents Susceptibility

**Figure 1:**
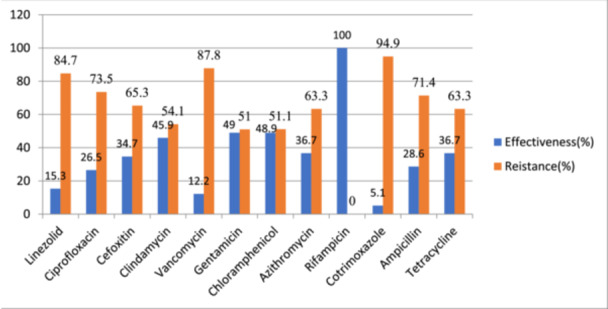
Antimicrobial agent's susceptibility pattern on strains of Staphylococcus *aureus* from healthcare workers at WUNEMMCSH, 2023.

The isolates' patterns of responsiveness to the tested antibiotics showed differing levels of tolerance and opposition. *S. aureus* was highly sensitive to most antibiotics tested. Drug effectiveness identified by *S. aureus* to rifampicin was 100%, followed by vancomycin (88%), linezolid (85%), and ciprofloxacin (73%). It is noted that 98% of *S. aureus* strains were in opposition to cotrimoxazole, and ampicillin resistance was 71% (Figure 1).

**Figure 2:**
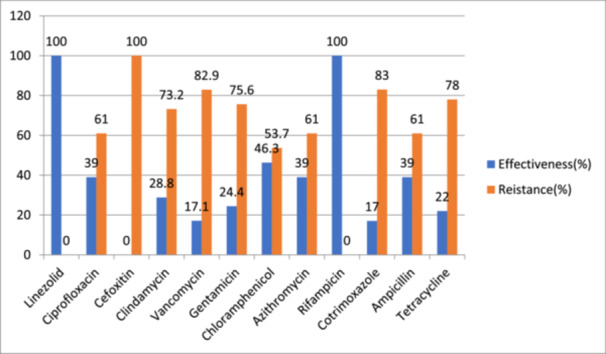
Antimicrobial agent's susceptibility pattern on strains of MRSA from healthcare workers at WUNEMMCSH, 2023.

In the case of MRSA, Sensitive drugs were Linezolid (100%), rifampicin (100%), vancomycin (82.9%), and gentamicin (75.6%). Strains of MRSA mentioned a considerable level of resistance (100%) to Cefoxitin, (83%) to ciprofloxacin, and 61% to Ciprofloxacin and ampicillin (Figure 2).

**Figure 3:**
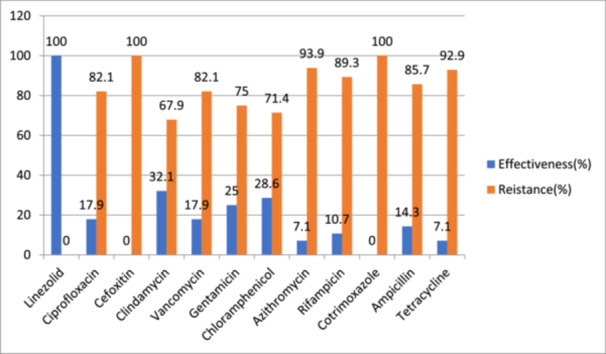
Antimicrobial agent's susceptibility pattern on strains of Biofilm‐producing MRSA from healthcare workers at WUNEMMCSH, 2023.

Antimicrobial susceptibility profiles of biofilm‐producing MRSA strains with different effectiveness and resistance were analyzed. Linezolid (100%), tetracycline (92.6%), rifampicin (89.3%), and vancomycin (82.1%) were highly sensitive to MRSA, also known that produces biofilm, whereas strains showed high degree of challenges within Cefoxitin, and Cotrimoxazole (100%), followed by Azithromycin (92.9%), Ampicillin (85.7%), and ciprofloxacin (82.1%) (Figure 3).

### 3.6 Multidrug Resistant Pattern of MRSA and Biofilm Producing MRSA

Multidrug‐resistant (MDR) profiles were detected in 65.8% (27/41) of anti‐methicillin *Staphylococcus aureus* and 22/28 (78.6%) of biofilm‐producing MRSA. The most typical MDR matches identified in MRSA or biofilm‐producing MRSA were opposite five groups of antibacterial: amoxicillin, cephalosporin, beta‐lactams, carpapinames, fluorquioles, and aminoglycosides. The relationship between organisms on biofilm producers that are resistant to multiple drugs and total MRSA isolates from healthcare workers was 22/41 (53.6%). Most of the isolates capable of forming biofilms exhibited substantial resistance to the antibiotic classes examined (Table 4).

**Table 4:** MRSA Multidrug resistance profile and biofilm‐producing MRSA isolates among healthcare workers of Wachemo University Nigist Ellen Mohammed Memorial Comprehensive Specialised Hospital, Central Ethiopia Regional State, 2023.

Resistance profile	MDR	Resistance profiles	Frequency, *n* (%)	SD
MRSA	R3	CIP,CHL,COT	5(12.1%)	0.41
	R3	CIP,COT,AMP	6(14.6%)	0.43
	R3	CHL,COT,AMP	7(17.1%)	0.44
	R4	CIP,CHL,COT,AMP	6(14.6%)	0.43
	R5	CIP,VAN,COT,AMP,CHL	3(7.3%)	0.35
Total			27/41(65.8%)	0.47
Biofilm‐producing MRSA	R3	CIP,CHL,COT	4(14.3%)	0.43
	R3	CIP,COT,AMP	6(21.4%)	0.41
	R3	VAN,AZM,AMP	5(17.9%)	0.31
	R4	CIP,COT,VAN,AMP	4(14.3%)	0.43
	R5	CIP,VAN,COT,AMP,CHL	3(10.7%)	0.35
Total			22/28(78.6%)	0.50

*Note:* “R3, R4, R5 indicate resistance to 3, 4, or 5 classes of antimicrobial agents.”

Abbreviations: AMP = Ampicillin, AZM = Azithromycin, CIP = Ciprofloxacin, CHL = Chloramphenicol, CLN = Clindamycin, CN = Gentamicin, COT = Cotrimoxazole, LIN = Linezolid, REF = Rifampicin, SD = Standard deviation, TE = Tetracycline, VAN = Vancomycin.

### 3.7 Factors Associated With Biofilm‐Producing MRSA Colonization

In the univariate analysis, three variables showed a *p*‐value of less than 0.25, which is prone to employing the multivariate model. Use of antibiotics previously for nasal infections with (AOR = 0.06% and 95% CI: 0.01–0.326), prior to hospitalization (length of stay in hospital for their illness purposes) with (AOR = 10.00% and 95% CI: 1.36–73.3), and HCWs with diabetes (AOR = 0.310, 95% CI: 0.078–1.238) was chosen as possible factors to analyze logistic regression using multiple variables. Likewise, an independent correlation between the suggested components, the multivariable logistic regression analysis, and nasal colonization due to biofilm‐producing MRSA was additionally evaluated at a cutoff point of *P* value below 0.05. Among them, previously used antibiotics for nasal infections and prior to hospitalization were associated independently with multiple‐variety logistic regression analysis, which revealed a notable correlation. The multivariable analysis found that use of antibiotics previously for nasal infections AOR = 0.06 (95% CI: 0.01–0.326) maintained its statistical significance as a factor linked to nasal colonization because biofilm forming MRSA. HCWs who had previously used antibiotics for nasal infections were approximately half times more likely to have nasal colonization of biofilm producing MRSA than those who had not previously used antibiotics due to biofilm producing MRSA. Similarly, prior to hospitalization (AOR = 10.00 (95% CI: 1.36–73.3) also remained statistically significant factor associated with nasal colonization due to biofilm producing MRSA. Prior to hospitalization (staying in hospital for long periods due to illness) were ten times more likely to have nasal colonization of biofilm producing MRSA than those who not were staying in hospital for long periods (Table 5).

**Table 5:** Assessment of bivariate and multivariate logistic regression on variables related to biofilm‐producing MRSA colonization among health workers at WUNEMMCSH, Central Ethiopia, 2023.

Variable		Biofilm‐producing MRSA		Crude odd ratio (95% CI)	*P*‐value	Adjusted odd ration (95% CI)	*P*‐value
		Present *N* (%)	Present *N* (%)				
Sex							
Male		11 (3.73)	98 (33.4)	1	1		1
Female		17 (17.4)	168 (57.1)	0.9 (0.4–2.00)	0.799	1.3 (0.2–7.3)	0.788
Age group							
25–34	185	5 (1.7)	180 (61.2)	11.35 (2.987–43.14)	0.00	2.1 (1.5‐ 12.8)	0.997
35–44	65	8 (2.7)	57 (19.4)	1.3 (0.4–4.65)	0.662	4.2 (1.2–14.5)	0.998
45–54	39	10 (3.4)	29 (9.9)	.58 (0.2–2.06)	0.407	2.7 (3.5–7.8)	0.998
55‐64	5	5 (1.7)	0 (0)	1	1	1	1
Total	294						
Work experience							
< 5		12 (4.1)	137 (46.6)	1.2 (0.12–10.3)	0.875	6.4 (3.8–23)	0.999
6‐10		8 (2.7)	37 (12.6)	1.4 (0.15–12.96)	0.76	1.3 (0.21–8.62)	0.999
11‐20		6 (2.1)	60 (20.4)	1.4 (0.15–13.65)	0.757	4.2 (0.62–21.1)	0.998
21‐30		1 (0.34)	27 (9.2)	2.8 (0.15–52)	0.478	0.57 (0.06–4.2)	0.998
> 30		1 (0.34)	5 (1.7)	1	1	0.3 (0.01–1.4)	0.276
Use of antibiotics previously for nasal infections							
Yes		16 (5.4)	87 (29.6)	0.238 (0.1–0.53)	0.000	0.06 (0.01–0.326)	0.001
No		12 (4.1)	179 (60.9)	1	1	1	1
Hand washing habit							
Always		13 (4.4)	147 (50.0)	1	1	1	1
Usually		13 (4.4)	76 (25.9)	0.44 (0.034–2.0)	0.295	1.85 (0.8–4.3)	.151
Sometimes		2 (0.68)	43 (14.6)	0.576 (0.124–2.67)	0.481	24.5 (5.2–114.5)	0.43
Use of antiseptics for hand rub							
Always		15 (5.1)	137 (46.6)	1	1		
Usually		11 (3.7)	100 (34.0)	0.84 (0.176–4.0)	0.829	18 (3.6–42.7)	0.996
Sometimes		2 (0.68)	29 (9.9)	1.69 (0.344–8.32)	0.517	4.3 (1.1–21.1)	0.997
Antiseptics used							
70% Alcohol only		9 (3.0)	119 (40.5)	1.96 (0.717–5.37)	.412	0.7 (0.23–1.8)	0.996
Sanitizer only		11 (3.74)	96 (32.65)	1.32 (0.499–3.477)	0.578	0.9 (0.3–1.9)	0.997
Both		8 (2.7)	51 (17.3)	1	1	1	1
Prior hospitalization							
Yes		13 (4.4)	51 (17.3)	0.343 (0.155–0.761)	0.008	10.00 (1.36–73.3)	0.024
No		15 (5.1)	215 (73.1)	1	1		
Diabetic mellitus							
Yes		10 (3.4)	27 (9.2)	0.237 (0.101–0.561)	0.001	0.310 (0.078–1.238)	0.097
No		18 (6.12)	239 (81.3)	1	1	1	1

## 4 Discussion

Our findings reveal a substantial rate of *Staphylococcus aureus* nasal carriage among healthcare workers, with a notable proportion of biofilm‐producing MRSA strains, emphasizing their critical role in persistent colonization, multidrug resistance, and potential nosocomial transmission*. Staphylococcus aureus* can colonize various body sites, including the intestinal tract, skin (epidermis), abdomen, oral cavity, genitals, axilla, and anterior nares (Wertheim et al. 2005). This study found a nasal carriage rate of *Staphylococcus aureus* of 33.4% among healthcare workers (HCWs), which is consistent with findings reported in different studies conducted worldwide. The result is comparable with a systematic review and meta‐analysis conducted in Ethiopia (26%) (Gobezie et al. 2025), and studies from Dessie, Ethiopia (28.8%) (Shibabaw et al. 2013), Gaza Strip (31.1%) (El Aila et al. 2017), Pakistan (38%) (Rashid et al. 2012), China (28.3%) (Chen et al. 2015), and Iran (28.7%) (Askarian et al. 2009). However, the prevalence observed in this study was higher than that reported in Wukiro‐Tigray, Northern Ethiopia (12%) (Legese et al. 2018), Harar, Eastern Ethiopia (15.6%) (Wolde et al. 2023), the Democratic Republic of Congo (16.5%) (De Boeck et al. 2015), and India (14%) (Malini et al. 2012). Conversely, it was lower than the prevalence reported in Hitherto, Nepal (52.2%) (Saud et al. 2023). These variations may be attributed to differences in microbiological methods (sampling procedures and culture techniques), local infection prevention and control guidelines, and screening periods for MRSA among HCWs.

In this study, the MRSA carriage rate was 13.9%, which is comparable to findings from Harar, Eastern Ethiopia (11.2%) (Wolde et al. 2023), Mekelle, Ethiopia (14.1%) (Shibabaw et al. 2013), Dessie (12.7%) (Mihret et al. 2013), Egypt (28%) (AT et al. 2010), and Pakistan (13.95%) (Rashid et al. 2012). However, higher prevalence rates were reported in Nigeria (39.9%) (Fadeyi 2010), Gaza Strip (25.5%) (El Aila et al. 2017), India (36%) (Bawankar et al. 2025) and Hitherto, Nepal (47.4%) (Saud et al. 2023). In contrast, lower MRSA prevalence was reported in Arba Minch, Ethiopia (7.4%) (Mekuriya et al. 2022), Nepal (10%) (Shakya et al. 2010), Assam, India (11.43%) (Rongpharpi et al. 2013), Turkey (6%), and Karnataka, India (8.3%) (Cesur and Cokça 2004; Vijaya et al. 2011). These discrepancies may be explained by differences in hospital infection prevention and control practices, healthcare services and resources, and antimicrobial surveillance policies (Wertheim et al. 2005).

A key finding of this study was that 68.3% of MRSA isolates were biofilm producers, which was significantly higher than non‐biofilm‐producing strains. This supports evidence that biofilm‐forming MRSA strains exhibit enhanced survival, persistence, and antimicrobial resistance, thereby complicating treatment outcomes. A study from Poland similarly demonstrated that MRSA isolates form biofilms more readily than MSSA isolates (Piechota et al. 2018). Biofilms protect bacteria against antibiotics, disinfectants, and host immune responses. Methicillin‐resistant strains have been reported to be nearly twice as common among biofilm producers compared to non‐producers (Rijal et al. 2020).

The resistance rates of *S. aureus* isolates to ampicillin (71.4%), cotrimoxazole (94.9%), and azithromycin (63.3%) are consistent with findings from Arba Minch, Ethiopia (Mekuriya et al. 2022), Dessie, Ethiopia (Shibabaw et al. 2014), and Nepal (Ansari et al. 2014). However, lower resistance rates to cotrimoxazole (33%) and gentamicin (27% and 25%) were reported in a systematic review conducted in Ethiopia (26%) (Gobezie et al. 2025), India (Malini et al. 2012) and Nepal (Khanal et al. 2015), respectively. These variations may be due to differences in antimicrobial resistance (AMR) monitoring programs and local antibiotic usage practices.

MRSA isolates in this study showed high resistance rates to cefoxitin (100%), cotrimoxazole (83%), ciprofloxacin (61%), and ampicillin (61%). However, high susceptibility was observed to linezolid and rifampicin (100% each), vancomycin (82.9%), tetracycline (78%), gentamicin (75.6%), and clindamycin (73.2%). Similarly, biofilm‐producing MRSA isolates showed high susceptibility to linezolid (100%), tetracycline (92.9%), rifampicin (89.3%), and vancomycin (82.1%), but complete resistance to cefoxitin and cotrimoxazole (100%), and high resistance to azithromycin (92.9%), ciprofloxacin (82.1%), and ampicillin (85.7%).

The high degree of resistance observed aligns with findings from Pakistan, where resistance to cefoxitin (100%), cotrimoxazole (83%), ciprofloxacin (61%), and ampicillin (61%) was reported (Rashid et al. 2012). Comparable antimicrobial susceptibility patterns were also observed in Nigeria (Fadeyi 2010), India (Diwakar et al. 2018), and Serbia (Cirkovic et al. 2015), although slight variations were noted.

Compared with Iranian healthcare worker studies, similar sensitivity patterns were reported for ciprofloxacin (66%), gentamicin (69%), and clindamycin (69%) (Tadesse et al. 2018). However, this study demonstrated higher ciprofloxacin resistance compared to previous Indian reports (20%) (M et al. 2013), In contrast, lower resistance rates were reported for azithromycin (19.6%), tetracycline (9.8%), gentamicin (3.9%), clindamycin (3.92%), and ciprofloxacin (3.92%) in Gaza Strip (El Aila et al. 2017) and India (Bawankar et al. 2025). The observed resistance patterns may be attributed to the overuse of antibiotics for various conditions and the replacement of susceptible strains by resistant ones within healthcare settings.

Most MRSA and biofilm‐forming MRSA isolates exhibited multidrug resistance (MDR), targeting five different antibiotic classes. Similar susceptibility patterns have been reported in Arba Minch, southern Ethiopia (Mekuriya et al. 2022), Dessie, Ethiopia (Shibabaw et al. 2014), and Tigray, northern Ethiopia (Legese et al. 2018). Increased multidrug resistance may result from genetic variation through mutation, horizontal transfer of resistance genes, overcrowding in hospital wards, and empirical antibiotic prescribing without culture and sensitivity testing (Rashid et al. 2012).

Among biofilm producers, 22 of 27 MRSA isolates (81.5%) were multidrug‐resistant. Most biofilm‐forming isolates demonstrated substantial resistance to the tested antibiotic classes. This finding is comparable to a study conducted among hospitalized patients elsewhere, where 85% of bacterial isolates were biofilm producers (Revdiwala et al. 2012). However, a lower proportion (56.4%) was reported at the University Hospital of Campania “Luigi Vanvitelli,” Naples, Italy (Folliero et al. 2021). Empirical antibiotic therapy should be guided by local epidemiological data on antimicrobial resistance patterns and biofilm production, particularly because these factors contribute to hospital‐acquired infections. The association between biofilm formation and antibiotic resistance highlights the need for further investigation into resistance mechanisms in biofilm‐producing strains.

In this study, prior antibiotic use for nasal and other infections was significantly associated with biofilm‐producing MRSA colonization. Healthcare workers who used antibiotics without microbiological confirmation were more likely to be colonized with biofilm‐producing MRSA in their anterior nares compared to those who received antibiotics based on microbiological evidence. This finding aligns with studies conducted in Egypt (Khairalla et al. 2017), the United States (Chamchod and Ruan 2012), France (Saadatian‐Elahi et al. 2013), and Taiwan (Pan et al. 2013). Biofilm‐producing MRSA contributes to increased pathogenicity, treatment failure, economic burden, and enhanced mechanisms of antibiotic resistance (Rather et al. 2021).

The study identified significant associations between biofilm‐producing MRSA colonization and two variables: prior antibiotic use and previous hospitalization (Table 5). Healthcare workers with recent antibiotic use were more likely to harbor biofilm‐producing MRSA, likely due to selective pressure and disruption of normal flora. Healthcare workers with recent hospital stays were more likely to harbor biofilm‐producing MRSA, likely due to increased exposure to nosocomial pathogens and potential immune compromise.

According to the current study, hospital stay was substantially linked to the spread of MRSA that produced biofilms. Healthcare professionals were highly likely to have biofilm‐producing MRSA colonization on their anterior nares prior to hospitalization. It's also consistent with investigations done in Tigray, Ethiopia (Legese et al. 2018), Tanzania (Geofrey et al. 2015), Iran (Askarian et al. 2009), and Taiwan (Wang et al. 2010). This may be because staying at the hospital causes nosocomial infections and reduces immunity, resulting in failure to combat pathogens (Geofrey et al. 2015).

Overall, these findings highlight the significant contribution of biofilm‐producing MRSA to multidrug resistance and emphasize the need to strengthen infection prevention and control strategies in healthcare settings.

### 4.1 Limitations of the Study

✓ Nasal colonization is caused by community or nosocomial strains that cannot be recognized.
✓ The particular species and variant types of MRSA strains that produce biofilm cannot be identified using more precise and accurate molecular detection methods.
✓ Additionally, phenotype‐ along with additional investigations are necessary for subsequent investigators to determine and elucidate the genetic mechanisms underlying antibiotic susceptibility.

## 5 Conclusions

The present study demonstrated a high prevalence of MRSA (13.9%) and an even higher proportion of biofilm‐producing MRSA (68.3%) among healthcare workers. A substantial percentage of these isolates exhibited multidrug resistance. Rifampicin, vancomycin, and linezolid are sensitive antimicrobials applied to MRSA medication and biofilm‐producing MRSA, whereas cotrimoxazole and ampicillin showed high resistance rates. The majority of the *MRSA* and biofilm‐producing MRSA isolates were MDR. Among MRSA isolates, 78.6% were MDR. Prior antibiotic use and previous hospitalization were significant predictors of biofilm‐producing MRSA colonization. These findings point to the urgent need for improved infection‐prevention practices and routine screening of healthcare workers to mitigate the risk of healthcare‐associated infections. Therefore, routine screening of healthcare workers combined with strengthened infection‐prevention and antimicrobial‐stewardship practices to control the spread of multidrug‐resistant, biofilm‐producing MRSA within the hospital environment. Finally, conducting future molecular studies is recommended to better understand strain diversity, biofilm mechanisms, and resistance patterns, thereby guiding more effective control measures.

## Author Contributions

Each author contributed significantly to the work reported, whether in the study design, execution, acquisition of information, data analysis, determination, or all of these areas. They also participated in the article's drafting, revision, or critical review, agreed on the journal to which the work was submitted, and agreed to take responsibility for every aspect of the work.

## Funding

The authors received no specific funding for this work.

## Ethics Statement

The authors have nothing to report.

## Consent

The aim of the investigation was described to healthcare workers about the nasal carriage pinpoints, and subsequently, a written description of the goal and methodology of the investigation was provided, and their consent was acquired.

## Conflicts of Interest

None declared.

## Supporting information


**Table 1:** Sampling frame for the healthcare workers who were found at the selected department in WUNEMMCSH, 2023. **Table 2:** Socio‐demographic characteristics of healthcare providers at WUNEMMCSH, Central Ethiopia, 2023. **Table 3:** Prevalence of biofilm forming MRSA isolates from healthcare workers at WUNEMMCSH 2023. **Table 4:** Antimicrobial susceptibility pattern of isolates from healthcare workers at WUNEMMCSH, 2023. **Table 5:** Multidrug resistance profile of nasal MRSA and biofilm producing MRSA isolates among healthcare workers of Wachemo University Nigist Ellen Mohammed memorial comprehensive specialized Hospital, Central Ethiopia Regional State, 2023. **Table 6:** Bivariate and Multivariate logistic regression analysis on factors associated with biofilm producing MRSA colonization among health workers at WUNEMMCSH Central Ethiopia, 2023.

## Data Availability

The paper contains all pertinent information, and upon request, the corresponding author can provide more information.
