# Supplementary material for: Nasal Carriage Rate of Biofilm Producing Methicillin Resistant Staphylococcus aureus and Its Associated Factors Among Health Care Workers at Hospital of Central Ethiopia
Source: Microbiologyopen. 2026 Mar 15;15(2):e70266. doi: 10.1002/mbo3.70266 (PMC13140737; doi:10.1002/mbo3.70266)
Supplement: Supplementary file 1 — Table 1: Sampling frame for the healthcare workers who were found at the selected department in WUNEMMCSH, 2023. Table 2: Socio‐demographic characteristics of healthcare providers at WUNEMMCSH, Central Ethiopia, 2023. Table 3: Prevalence of biofilm forming MRSA isolates from healthcare workers at WUNEMMCSH 2023. Table 4: Antimicrobial susceptibility pattern of isolates from healthcare workers at WUNEMMCSH, 2023. Table 5: Multidrug resistance profile of nasal MRSA and biofilm producing MRSA isolates among healthcare workers of Wachemo University Nigist Ellen Mohammed memorial comprehensive specialized Hospital, Central Ethiopia Regional State, 2023. Table 6: Bivariate and Multivariate logistic regression analysis on factors associated with biofilm producing MRSA colonization among health workers at WUNEMMCSH Central Ethiopia, 2023. [file MBO3-15-e70266-s001.docx]

**Table 1**: Sampling frame for the healthcare workers who were found at the selected department in WUNEMMCSH, 2023

| **Departments** | **Number of healthcare workers** | |
| --- | --- | --- |
|  | Total (N_i_ ) | Sample (ni) |
| Medical | 74 | 28 |
| Pediatrics | 98 | 33 |
| Gynecology–Obstetrics | 110 | 31 |
| Laboratory | 72 | 25 |
| OPD | 142 | 47 |
| Pharmacy | 58 | 21 |
| Surgical | 86 | 30 |
| Orthopedics | 78 | 22 |
| ICUs | 110 | 22 |
| Emergency | 156 | 35 |
| Total | 984 | 294 |

- Where ni: is the sample for the strata, N_i:_  is the total population of each stratum

**Table 2: Socio-demographic characteristics of healthcare providers at WUNEMMCSH, Central Ethiopia, 2023**

| S.No  **1** | Variables | Frequency | Percent |
| --- | --- | --- | --- |
|  | Sex  Male  Female | 109  185 | 37.1  62.9 |
| 2 | Age group  25–34  35–44  45–54  55-64 | 185  65  39  5 | 62.9  22.1  13.3  1.7 |
| 3 | Work experience in years  >1/2 - 5  6-10  11-20  21-30  >30 | 149  45  66  28  6 | 50.6  15.3  22.5  9.5  2.1 |
| 4 | Profession  Medical Doctor  Nurse  Midwife  Laboratory  Pharmacy | 61  119  68  25  21 | 20.7  40.5  23.1  8.5  7.1 |
| **5** | Working site  Medical  Surgical  Pediatric  Gynecology-obstetrics  Laboratory  Outpatient department  ICU  Pharmacy  Emergency department  Orthopedic | 28  30  33  31  25  47  22  21  35  22 | 9.5  10.2  11.2  10.5  8.5  16.0  7.4  7.1  12.0  7.4 |
| 6 | Use of antibiotics before one week of swab collection  Yes  No | 103  191 | 35.1  64.9 |
| 7 | Use of Mask  Yes  No | 240  54 | 81.6  18.4 |
| 8 | Hand washing habit after and before patient attendance  Always  Usually  Not at all | 160  89  45 | 54.4  30.3  15.3 |
|  | Use of antiseptics for hand rub  Always  Usually  Not at all | 152  111  31 | 51.7  37.8  10.5 |
|  | Antiseptics used after and before patient touch  70% Alcohol only  Sanitizer only  Both | 128  107  59 | 43.5  36.4  20.1 |
| 11 | Prior hospitalization  Yes  No | 64  230 | 21.8  78.2 |
| 12 | Diabetic mellitus  Yes  No | 37  257 | 12.6  87.4 |

**Table 3: Prevalence of biofilm forming MRSA isolates from healthcare workers at WUNEMMCSH 2023**

| Bacteria isolates | Biofilm production | | Total |
| --- | --- | --- | --- |
|  | Biofilm producer | Non-biofilm producer |  |
| MRSA | 28(68.3%),  95% CI;(47.6-89.0) | 13(31.7%) | 41(41.8%) |
| MSSA | 27(47.4%) | 30(52.6%) | 57(58.2%) |
| Total | 55(56.1%) | 43(43.9%) | 98(100.0%) |

- MRSA: Methicillin-resistant *Staphylococcus aureus,* MSSA: Methicillin-sensitive *Staphylococcus aureus*

**TABLE 4: Antimicrobial susceptibility pattern of isolates from healthcare workers at WUNEMMCSH, 2023**

| **Bacterial isolates** | Patterns | **Antimicrobials tested** | | | | | | | | | | | |
| --- | --- | --- | --- | --- | --- | --- | --- | --- | --- | --- | --- | --- | --- |
|  |  | **LIN** | **CIP** | **CXT** | **CLN** | **VAN** | **CN** | **CHL** | **AZM** | **REF** | **COT** | **AMP** | **TE** |
| *S. aureus*  N = 98 | S(n) | 83 | 72 | 64 | 45 | 86 | 50 | 48 | 36 | 98 | 5 | 28 | 62 |
|  | % | 84.7 | 73.5 | 65.3 | 45.9 | 87.8 | 51 | 48.9 | 36.7 | 100 | 5.1 | 28.6 | 63.3 |
|  | R( n) | 15 | 26 | 34 | 53 | 12 | 48 | 50 | 62 | 0 | 93 | 70 | 36 |
|  | % | 15.3 | 26.5 | 34.7 | 54.1 | 12.2 | 39 | 51.1 | 63.3 | 0 | 94.9 | 71.4 | 36.7 |
| MRSA  N=41 | S( n) | 41 | 16 | 0 | 30 | 34 | 31 | 19 | 25 | 41 | 7 | 16 | 32 |
|  | % | 100 | 39 | 0 | 73.2 | 82.9 | 75.6 | 46.3 | 61 | 100 | 17 | 39 | 78 |
|  | R( n) | 0 | 25 | 41 | 11 | 7 | 10 | 22 | 17 | 0 | 34 | 25 | 9 |
|  | % | 0 | 61 | 100 | 28.8 | 17.1 | 24.4 | 53.7 | 39 | 0 | 83 | 61 | 22 |
| Biofilm producing MRSA  N=28 | S (n) | 28 | 5 | 0 | 19 | 23 | 21 | 8 | 2 | 25 | 0 | 4 | 26 |
|  | % | 100 | 17.9 | 0 | 67.9 | 82.1 | 75 | 28.6 | 7.1 | 89.3 | 0 | 14.3 | 92.9 |
|  | R (n) | 0 | 23 | 28 | 9 | 5 | 7 | 20 | 26 | 3 | 28 | 24 | 2 |
|  | % | 0 | 82.1 | 100 | 32.1 | 17.9 | 25 | 71.4 | 92.9 | 10.7 | 100 | 85.7 | 7.1 |

**Key:** AMP=Ampicillin, CHL=Chloramphenicol, CIP=Ciprofloxacin, REF=Rifampicin, CLN=Clindamycin, AZM=Azithromycin, CN=Gentamicin, LIN=Linezolid, COT= Cotrimoxazole TE=Tetracycline, VAN=Vancomycin, CXT=Cefoxitin,S= Sensitive, R= Resistant

**Table 5: Multidrug resistance profile of nasal MRSA and biofilm producing MRSA isolates among healthcare workers of Wachemo University Nigist Ellen Mohammed memorial comprehensive specialized Hospital, Central Ethiopia Regional State, 2023.**

| Resistance profile | MDR | Resistance profiles | **Frequency,**  **n (%)** |
| --- | --- | --- | --- |
| MRSA | R3 | CIP,CHL,COT | 5(12.1%) |
|  | R3 | CIP,COT,AMP | 6(14.6%) |
|  | R3 | CHL,COT,AMP | 7(17.1%) |
|  | R4 | CIP,CHL,COT,AMP | 6(14.6%) |
|  | R5 | CIP ,VAN,COT,AMP,CHL | 3(7.3%) |
| Total |  |  | 27/41(65.8%) |
| Biofilm producing MRSA | R3 | CIP,CHL,COT | 4(14.3%) |
|  | R3 | CIP,COT,AMP | 6(21.4%) |
|  | R3 | VAN,AZM,AMP | 5(17.9%) |
|  | R4 | CIP,COT,VAN,AMP | 4(14.3%) |
|  | R5 | CIP ,VAN,COT,AMP,CHL | 3(10.7%) |
| Total |  |  | 22/28(78.6%) |

**Key:** AMP=Ampicillin, CHL=Chloramphenicol, CIP=Ciprofloxacin, REF=Rifampicin, CLN=Clindamycin, AZM=Azithromycin, CN=Gentamicin, LIN=Linezolid, COT= Cotrimoxazole TE=Tetracycline, VAN=Vancomycin,

**Table 6: Bivariate and Multivariate logistic regression analysis on factors associated with biofilm producing MRSA colonization among health workers at WUNEMMCSH Central Ethiopia, 2023**

| Variable | | Biofilm producing MRSA | | COR (95% CI) | *P*-value | AOR (95% CI) | *P*-value |
| --- | --- | --- | --- | --- | --- | --- | --- |
|  |  | Present N (%) | Absent N (%) |  |  |  |  |
| **Sex** | |  |  |  |  |  |  |
| Male | | 11(3.73) | 98(33.4) | 1 | 1 |  | 1 |
| Female | | 17(17.4) | 168(57.1) | 0.9(0.4-2.00) | 0.799 | 1.3(0.2-7.3) | 0.788 |
| **Age group** | |  |  |  |  |  |  |
| 25–34 | 185 | 5(1.7) | 180(61.2) | 11.35(2.987-43.14) | 0.00* | 2.1(1.5- 12.8) | 0.997 |
| 35–44 | 65 | 8(2.7) | 57(19.4) | 1.3(0.4-4.65) | 0.662 | 4.2(1.2-14.5) | 0.998 |
| 45–54 | 39 | 10(3.4) | 29(9.9) | .58(0.2-2.06) | 0.407 | 2.7(3.5-7.8) | 0.998 |
| 55-64 | 5 | 5(1.7) | 0(0) | 1 | 1 | 1 | 1 |
| Total | 294 |  |  |  |  |  |  |
| **Work experience** | |  |  |  |  |  |  |
| <5 | | 12(4.1) | 137(46.6) | 1.2(0.12-10.3) | 0.875 | 6.4(3.8-23) | 0.999 |
| 6-10 | | 8(2.7) | 37(12.6) | 1.4(0.15-12.96) | 0.76 | 1.3(0.21-8.62) | 0.999 |
| 11-20 | | 6(2.1) | 60(20.4) | 1.4(0.15-13.65) | 0.757 | 4.2(0.62-21.1) | 0.998 |
| 21-30 | | 1(0.34) | 27(9.2) | 2.8(0.15-52) | 0.478 | 0.57(0.06-4.2) | 0.998 |
| >30 | | 1(0.34) | 5(1.7) | 1 | 1 | 0.3(0.01-1.4) | 0.276 |
| **Use of antibiotics previously for nasal infections** | |  |  |  |  |  |  |
| Yes | | 16(5.4) | 87(29.6) | 0.238(0.1-0.53) | 0.000* | 0.06(0.01-0.326) | 0.001* |
| No | | 12(4.1) | 179(60.9) | 1 | 1 | 1 | 1 |
| **Hand washing habit** | |  |  |  |  |  |  |
| **Always** | | 13(4.4) | 147(50.0) | 1 | 1 | 1 | 1 |
| **Usually** | | 13(4.4) | 76(25.9) | 0.44(0.034-2.0) | 0.295* | 1.85(0.8-4.3) | .151 |
| Sometimes | | 2(0.68) | 43(14.6) | 0.576(0.124-2.67) | 0.481 | 24.5(5.2-114.5) | 0.43 |
| **Use of antiseptics for hand rub** | |  |  |  |  |  |  |
| Always | | 15(5.1) | 137(46.6) | 1 | 1 |  |  |
| Usually | | 11(3.7) | 100(34.0) | 0.84(0.176-4.0) | 0.829 | 18(3.6-42.7) | 0.996 |
| Sometimes | | 2(0.68) | 29(9.9) | 1.69(0.344-8.32) | 0.517 | 4.3(1.1-21.1) | 0.997 |
| **Antiseptics used** | |  |  |  |  |  |  |
| 70% Alcohol only | | 9(3.0) | 119(40.5) | 1.96(0.717-5.37) | .412 | 0.7(0.23-1.8) | 0.996 |
| Sanitizer only | | 11(3.74) | 96(32.65) | 1.32(0.499-3.477) | 0.578 | 0.9(0.3-1.9) | 0.997 |
| Both | | 8(2.7) | 51(17.3) | 1 | 1 | 1 | 1 |
| **Prior hospitalization** | |  |  |  |  |  |  |
| Yes | | 13(4.4) | 51(17.3) | 0.343(0.155-0.761) | 0.008* | 10.00(1.36-73.3) | 0.024* |
| No | | 15(5.1) | 215(73.1) | 1 | 1 |  |  |
| **Diabetic mellitus** | |  |  |  |  |  |  |
| Yes | | 10(3.4) | 27(9.2) | 0.237(0.101-0.561) | 0.001* | 0.310(0.078-1.238) | 0.097 |
| No | | 18(6.12) | 239(81.3) | 1 | 1 | 1 | 1 |
